# Reproducibility of the airway response to an exercise protocol standardized for intensity, duration, and inspired air conditions, in subjects with symptoms suggestive of asthma

**DOI:** 10.1186/1465-9921-11-120

**Published:** 2010-09-01

**Authors:** Sandra D Anderson, David S Pearlman, Kenneth W Rundell, Claire P Perry, Homer Boushey, Christine A Sorkness, Sara Nichols, John M Weiler

**Affiliations:** 1 Department of Respiratory & Sleep Medicine, 11 West, Royal Prince Alfred Hospital, Missenden Road, Camperdown NSW 2050, Australia; 2 Sydney Medical School, University of Sydney, NSW 2006, Australia; 3 Colorado Allergy and Asthma Centers, Suite 150/125 Rampart Way, Denver CO 80230- 6405, USA; 4 Professor of The Basic Sciences, The Commonwealth Medical College, 150 North Washington Avenue, Scranton PA, PA 18503-1843, USA; 5 Department of Respiratory & Sleep Medicine, 11 West, Royal Prince Alfred Hospital, Missenden Road, Camperdown NSW 2050, Australia; 6 Asthma Clinical Research Center, University of California, San Francisco CA 90089, USA; 7 Department of Medicine, Allergy and Asthma Clinical Research, University of Wisconsin, Madison, WI 53705, USA; 8 CompleWare Corporation, PO Box 3090, North Liberty, IA 52317, USA; 9 Department of Internal Medicine, University of Iowa, Iowa City, IA52242, USA

## Abstract

**Background:**

Exercise testing to aid diagnosis of exercise-induced bronchoconstriction (EIB) is commonly performed. Reproducibility of the airway response to a standardized exercise protocol has not been reported in subjects being evaluated with mild symptoms suggestive of asthma but without a definite diagnosis. This study examined reproducibility of % fall in FEV_1 _and area under the FEV_1 _time curve for 30 minutes in response to two exercise tests performed with the same intensity and duration of exercise, and inspired air conditions.

**Methods:**

Subjects with mild symptoms of asthma exercised twice within approximately 4 days by running for 8 minutes on a motorized treadmill breathing dry air at an intensity to induce a heart rate between 80-90% predicted maximum; reproducibility of the airway response was expressed as the 95% probability interval.

**Results:**

Of 373 subjects challenged twice 161 were positive (≥10% fall FEV_1 _on at least one challenge). The EIB was mild and 77% of subjects had <15% fall on both challenges. Agreement between results was 76.1% with 56.8% (212) negative (< 10% fall FEV_1_) and 19.3% (72) positive on both challenges. The remaining 23.9% of subjects had only one positive test. The 95% probability interval for reproducibility of the % fall in FEV_1 _and AUC_0-30 _min was ± 9.7% and ± 251% for all 278 adults and ± 13.4% and ± 279% for all 95 children. The 95% probability interval for reproducibility of % fall in FEV_1 _and AUC_0-30 min _for the 72 subjects with two tests ≥10% fall FEV_1 _was ± 14.6% and ± 373% and for the 34 subjects with two tests ≥15% fall FEV_1 _it was ± 12.2% and ± 411%. Heart rate and estimated ventilation achieved were not significantly different either on the two test days or when one test result was positive and one was negative.

**Conclusions:**

Under standardized, well controlled conditions for exercise challenge, the majority of subjects with mild symptoms of asthma demonstrated agreement in test results. Performing two tests may need to be considered when using exercise to exclude or diagnose EIB, when prescribing prophylactic treatment to prevent EIB and when designing protocols for clinical trials.

## Background

Exercise is a widely recognised stimulus for provoking transient airway narrowing. Exercise-induced bronchoconstriction (EIB) is the term used to describe this phenomenon. The most commonly used measure to express severity of EIB is the post-exercise fall in forced expiratory volume in one second (FEV_1_), as a percentage of the pre-exercise value [1]. A ≥10% fall in FEV_1 _is reported to provide the best discrimination between asthmatic and normal responses in laboratory based running tests [2]. It is also the value suggested as the cut off for a positive test in the ATS and ERS guidelines for testing for EIB [3,4]. A second index of EIB severity is the area under the % fall in FEV_1 _time curve (AUC_0-30 min_), which summarizes the extent and duration of bronchoconstriction. This second index is used to assess the benefit of medications that enhance recovery to a greater extent than their benefit on the immediate post exercise fall in FEV_1 _[5]. The AUC_0-30 min _reflects the contribution of the numerous mediators involved in EIB [6,7].

EIB commonly occurs in people with clinically recognized asthma [8] and has been reported in school children, elite athletes, and military recruits without other clinical signs and symptoms of asthma [9-11]. EIB is often the first indication of asthma [12] so it is important to diagnose and then treat underlying asthma recognized by exercise intolerance. We recently studied and reported a large number of adults and children with signs and symptoms suggestive of asthma but without a definitive diagnosis [13]. The study investigated sensitivity and specificity of airway responsiveness to methacholine and mannitol to identify EIB and a physician diagnosis of asthma [13]. The study examined duplicate controlled exercise challenges in 373 subjects and the data provided an opportunity to examine reproducibility of the airway response to exercise in the type of individual most likely to be referred for exercise testing for EIB.

Exercise testing to identify EIB in the laboratory is affected by the type of exercise, intensity and duration of exercise, inspired air conditions, baseline lung function and time since last medication or exercise. This paper reports the reproducibility of the % fall in FEV_1 _and AUC_0-30 min _in response to an exercise protocol that carefully controlled these variables.

## Methods

### Subjects: Inclusion/Exclusion Criteria

Subjects were enrolled if they were aged 6-50 years with a BMI of <35, and reported signs and symptoms suggestive of asthma according to the National Institute of Health (NIH) Questionnaire [14]. They were required to have an FEV_1 _≥70% of the predicted value at the Screening Visit [15,16]. Subjects were required to have a National Asthma Education and Prevention Program (NAEPPII) asthma severity score of Step 1 with neither a firm diagnosis of asthma nor an exclusion of the diagnosis of asthma. Step 1 of NAEPPII is the mildest and is defined as symptoms ≤2 times per week, asymptomatic and normal peak expiratory flow measurements between exacerbations, exacerbations from only a few hours to a few days, night time symptom frequency of ≤ 2 times per month, FEV_1 _or PEF ≥80% predicted and PEF variability ≤20%.

Subjects were excluded from participation if they: had any known other pulmonary disease; had smoked more than 1 cigarette per week within the past year or had a ≥10 pack year smoking history; had a respiratory tract infection within the previous 4 weeks; had been skin test positive to aeroallergens that were present in the environment during the time of enrolment and reported worsening of symptoms when exposed to these aeroallergens during the study; had been diagnosed at the Screening Visit as definitively (95 to 100% likelihood) having or not having asthma; had clinically significantly abnormal chest x-ray or ECG; or had failed to observe washout time of medications that would interfere with exercise (including, but not limited to, no use of corticosteroids within 4 weeks of the Screening Visit).

**Figure 1:**
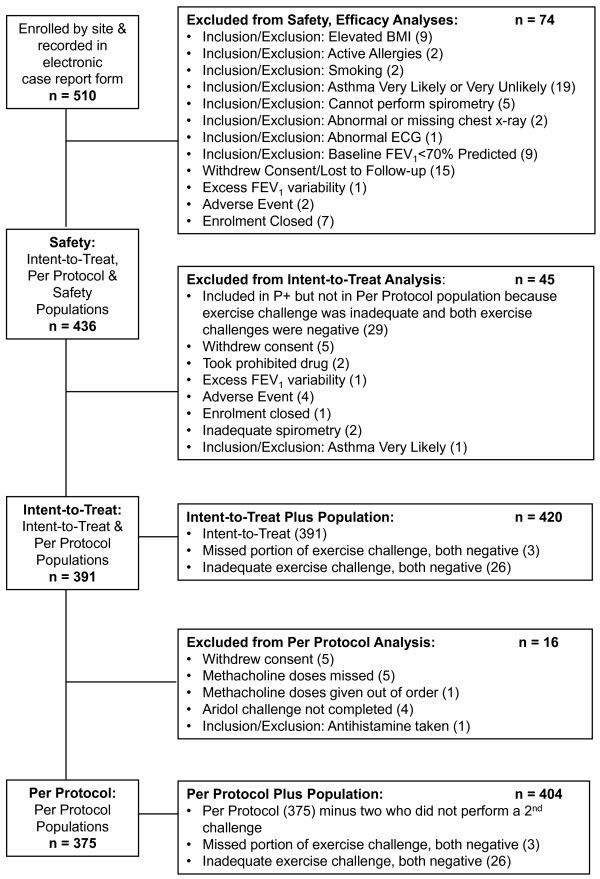
**Subject Disposition**. Reproduced from *Respiratory Research *2009, 10:4 (23 January 2009) with the permission of the authors.

The disposition of the study population is given in Figure 1. The data presented are from the 375 subjects in the per protocol population that included all subjects with no major protocol violations previously reported [13]. Of the 375 subjects, two completed only one exercise challenge leaving 373 who completed two exercise tests; there were 95 children and 278 adults.

### Procedures

The protocol was approved by institutional review boards and performed at 25 sites in the USA. Each subject or parent gave written informed consent or assent for minors <18 years of age. At screening the following were assessed: eligibility; demography; medical history; medications; spirometry with reversibility (following 360 mcg of albuterol/salbutamol from a pressurised metered dose inhaler); and allergy skin-prick testing to 10 common allergens (positive test taken as a wheal size ≥3 mm of the control). The NIH NAEPPII Questionnaire was administered and a score was assigned.

Exercise was performed on two separate occasions beginning 1 - 4 days after the screening visits and within 2 hrs of the same time of day. Medication withholding was confirmed (Table 1), and spirometry was measured to determine consistency with values obtained at screening as previously described [13]. The exercise was performed on consecutive visits (2 and 3) with the second challenge being in 1 - 4 days after the first. FEV_1 _needed to be >70% predicted and within 15% of FEV_1 _at screening in order for an exercise challenge to be performed.

**Table 1:** Required medication withholding periods for medications before exercise tests.

	Factor	Withholding Period
Inhaled agents	Short acting bronchodilators (isoproterenol, isoetharine, metaproterenol, albuterol, levalbuterol, terbutaline) (e.g. Proventil^® ^or Ventolin^®^)	8 hr
	
	Inhaled anticholinergics or combination products (e.g. Atrovent^® ^or Combivent^®^)	1 week
	
	Long acting inhaled bronchodilators (salmeterol, formoterol) (e.g. Serevent^® ^or Foradil^®^)	2 weeks
	
	Inhaled corticosteroid/long acting inhaled bronchodilator combination (e.g. Advair^®^)	4 weeks

**Oral bronchodilators**	Theophylline	24 hr
	
	Intermediate theophylline	48 hr
	
	Long acting theophylline	48 hr
	
	Standard β-agonist tablets	24 hr
	
	Long acting β-agonist tablets	48 hr

**Corticosteroids**	There is no washout for topical corticosteroids applied to skin unless they are high potency steroids	4 weeks

**Other medications**	Hydroxyzine, cetirizine (and other antihistamines)	72 hr
	
	Tiotropium bromide	72 hr
	
	Nasals corticosteroids	1 week
	
	β-blockers	1 week
	
	Cromolyn sodium	2 weeks
	
	Nedocromil	2 weeks
	
	Leukotriene modifiers	6 weeks

**Foods**	Coffee, tea, cola drinks, chocolate (caffeinated foods)	12 hr

**Strenuous exercise or exposure to cold air to a level that would be expected to interfere with challenges**		12 hr

**Tobacco**		6 hr

### Exercise protocol

Exercise was performed by running on a motorized treadmill while breathing medical grade dry air (20-25°C) from a reservoir (Douglas Bag) via a two-way non-rebreathing valve [17]. Subjects began by walking then running with the treadmill speed at 2.5 mph with 2.5% incline. Speed and incline were increased over 2 minutes so that heart rate (HR) reached 80-90% of predicted maximum (220-age) and then was maintained for 6 minutes for a total duration of 8 minutes. This intensity aimed to achieve a ventilation rate between 14 and 21 times FEV1 L values that represent between 40 and 60% of maximum predicted ventilation (35 × FEV_1_) [18]. The challenge could be stopped at any time. HR was monitored during and for 30 min after exercise.

FEV_1 _and FVC were measured before and FEV_1 _(not FVC) was measured 5, 10, 15, and 30 minutes after exercise. The % fall in FEV_1 _was calculated by subtracting the lowest value recorded after exercise taking the best of two acceptable attempts at each time point, from the value measured immediately before exercise, expressed as a percentage of the pre-exercise value. Values were not rounded; a 9.99% fall was considered negative. A subject was deemed positive if there was a fall of ≥10% in FEV_1 _at one time point on at least one of the two exercise challenges [3,4]. Values are reported as mean and standard deviation (SD). Values for FEV_1 _post-exercise that remained higher than the pre-exercise value were censored as 0% falls. The AUC_0-30 min _was calculated by the trapezoidal method [19] and expressed as % fall in FEV_1 _min^-1^.

Spirometry data were captured by using ClinDataLink^® ^(CDL) (CompleWare Corporation, North Liberty, IA) and met or exceeded the requirements proposed by American Thoracic Society/European Respiratory Society Joint Statement [20]. Calibration was verified each day at three flow rates before use. WebCDL^® ^software displayed an electronic record of the volume-time curves, flow-volume displays, and flow-time displays.

An estimate was made of ventilation in the 2^nd ^and 6^th ^minutes of exercise based on the relationship between speed and incline of treadmill and oxygen consumption in ml [21]. The ventilatory equivalent was estimated as 27 L per L of VO_2 _[22], and ventilation was expressed as % of maximum voluntary ventilation (MVV). The estimate of oxygen consumption in mls was:

1.262*weight*(3.5 + (5.36*speed) + (0.24*speed*incline)) for running

1.262*weight*(3.5 + (2.68*speed) + (0.48*speed*incline)) for walking.

Weight is expressed in kilograms and speed is expressed in miles per hour. Three miles per hour was taken to be running.

### Statistical Analysis

Reproducibility of the exercise test response was illustrated using a Bland-Altman-type plot [23] and calculated using the method of Chinn [24]. In brief, the standard deviation of a single measurement was calculated by dividing the standard deviation of the differences in % fall in FEV_1 _values between the two tests (i.e. 7.6 for the whole group) by the square root of 2 giving a 5.4% fall, from which we calculated a 95% probability interval of ± 10.8%. This interval defines a 95% probability that the difference between any single measurement and the true value for the subject is within that range. This gives information about variability of the response that can be expected in an individual with repeated testing.

## Results

### Demography

For the per protocol population (n = 375): females comprised 51.5%; subjects were 76.3% Caucasian, 8.3% Hispanic and 8.5% Black; subjects had near-normal baseline spirometry (Table 2); and 7.2% responded positively to a bronchodilator with ≥12% and ≥200 ml increase in FEV_1 _above baseline. The characteristics of the 95 children and 278 adults are summarised in Table 2. The mean NAEPPII asthma score was 1.22 (SD 0.52) for the adults and 1.21 (0.48) for the children. Positive skin tests to at least one allergen were seen in 78% of the adults and children.

**Table 2:** Anthropometric data, forced expiratory volume in one second, and smoking history in the per protocol population.

Children								
**N = 95**	**Age (yr)**	**BMI**	**FEV_1 _(L)**	**% Pred FEV_1_**	**% Rise Post BD FEV_1 _(L)**	**Pack Yrs** **N = 1**	**Ht (cm)**	**Wt (kg)**

**Mean**	13.0	21.5	2.83	94.2	6.9	0.43	157.6	54.9

**SD**	3.0	4.3	0.92	12.5	12.8		16.7	18.2

**Range**	6-17	13.4-33.1	1.15-5.15	63.7-127.4	0-115		118-192	20-102

**Median**	14	21.3	2.69	92.2	4.4		158	54.9

**Adults**								

**N = 278**	**Age (yr)**	**BMI**	**FEV_1 _(L)**	**% Pred FEV_1_**	**% Rise Post BD FEV_1 _(L)**	**Pack Yrs** **N = 44**	**Ht (cm)**	**Wt (kg)**

**Mean**	28.2	25.3	3.49	93.4	5.1	3	170.7	74.2

**SD**	8.8	4.1	0.71	10.2	5.8	2.9	9.7	15.7

**Range**	18-50	14.7-34.9	1.97-5.62	70.3-140.1	0-51.5	0-9	150-204	38-135

**Median**	25	25.0	3.38	93.3	3.99	2.5	170	72.3

### Reproducibility of the Response

The 373 subjects who completed two exercise challenges did so within 2.6 ± 3.2 (median 2) days. The agreement for exercise response was 76.1% with 56.8% (212) negative and 19.3% (72) positive on both challenges. Seventy-two, 34, and 19 of the 373 subjects had FEV_1 _falls of ≥10%, ≥15% ≥20%, respectively on both exercise challenges.

**Figure 2:**
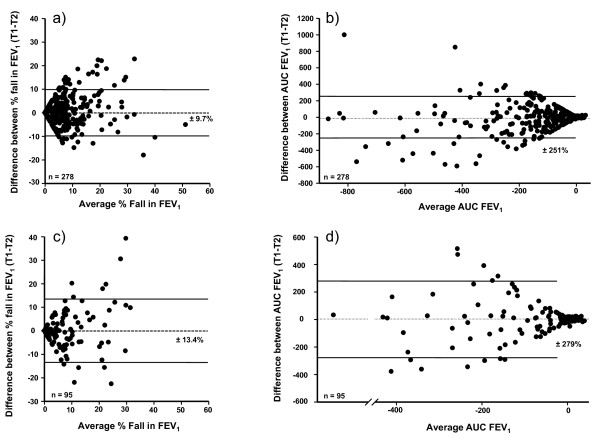
**Reproducibility of the % fall in FEV_1 _and area under the FEV_1 _curve following exercise**. The difference between values for % fall FEV_1 _and AUC_0-30 min _% fall FEV_1 _per min on the two exercise challenges in relation to the average value for the two challenges in adults (a and b) and children (c and d). The interval defines the 95% probability that the difference between a single measurement and the true value for the subject is within that range.

**Figure 3:**
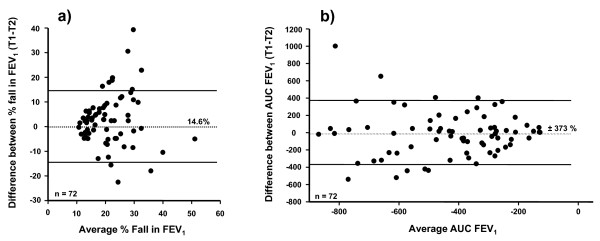
**Reproducibility of the % fall in FEV_1 _and area under the FEV_1 _curve following exercise in subjects positive on both occasions**. The difference between values for a) % fall in FEV_1_; and b) AUC_0-30 min _on the two challenges in relation to the average value on the two challenges for those who had a fall in FEV_1 _≥10% on both challenges. The interval defines the 95% probability that the difference between a single measurement and the true value for the subject is within that range.

The reproducibility (95% probability value) of the % fall in FEV_1 _and the AUC % fall in FEV_1 _min^-1 ^for the whole group and for adults and children separately are given in Table 3, together with mean and highest falls in FEV_1_. The variation for the response in all the adults and all the children is illustrated in Figures 2 and in Figures 3a and 3b for those with ≥10% fall in FEV_1 _on both tests.

**Table 3:** Values for the 95% probability interval for % fall in FEV_1 _and AUC, highest % fall in FEV_1_, the associated AUC, mean % fall FEV_1 _and the SD of the difference between two tests shown for Groups and for different NAEPP values.

	%Fall FEV_1_	AUC % fall FEV_1 _min^-1^	mean ± SD Highest % Fall FEV_1_	mean ± SD AUC % fall FEV_1 _min^-1^	Mean % fall FEV_1 _two tests	SD difference two tests % fall FEV_1_
**Whole Group** **n = 373**	± 10.8%	± 259%	10.95% ± 9.4	-221% ± 221	8.2	7.6

**Adults** **n = 278**	± 9.7%	± 251%	10.4% ± 8.9	-212% ± 214	7.9	6.9

**Children** **n = 95**	± 13.4%	± 279%	12.6% ± 10.5	-249% ± 239	9.3	9.5

**2 tests ≥ 10%** **n = 72**	± 14.6%	± 373%	24.7% ± 9.7	-525% ± 245	20.8	10.3

**2 tests ≥ 15%** **n = 34**	± 12.2%	± 411%	29.4 ± 8.5	-613% ± 259	25.9	8.6

**2 tests ≥ 20%** **n = 19**	± 14.3	± 470%	34.0 ± 8.2	-707% ± 246	30.1	10.1

**1 test ≥ 10%** **n = 89**	± 15.7	± 370%	14.3 ± 4.8	-289% ± 151	9.4	11.1

**2 tests <10%** **n = 212**	± 5.2%	± 117%	4.9% ± 2.9	-89% ± 75	3.5	3.7

**2 tests < 15%** **n = 288**	± 7.1%	± 168%	6.8% ± 4.2	-132% ± 107	4.9	5.0

**NAEPP Scores**						

**NAEPP = 1** **n = 309**	±10.7%	± 252%	10.7% ± 9.2	-206% ± 211	8.1	7.5

**NAEPP > 1** **n = 64**	± 11.4%	± 289%	12.0% ± 10.1	-249% ± 248	9.1	8.1

**NAEPP = 2** **n = 48**	± 10.3%	± 284%	10.3% ± 8.9	-228% ± 252	7.8	7.3

**NAEPP = 3** **n = 16**	± 14.6%	± 312%	17.1 ± 11.8	-313% ± 235	12.9	10.3

**Figure 4:**
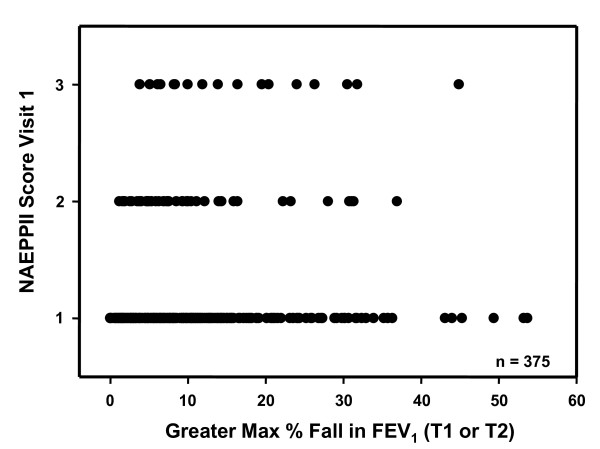
**% fall in FEV_1 _in relation to NAEPPII severity score**. Individual values for the maximum % fall in FEV_1 _after exercise in relation to the NAEPPII severity grading for asthma.

The reproducibility of the exercise response in relation to the different NAEPPII scores is given in Table 3. There was no relationship between the NAEPII score and the severity of the response to exercise expressed as the % fall in FEV_1 _after exercise (Figure 4).

### Exercise Response

**Figure 5:**
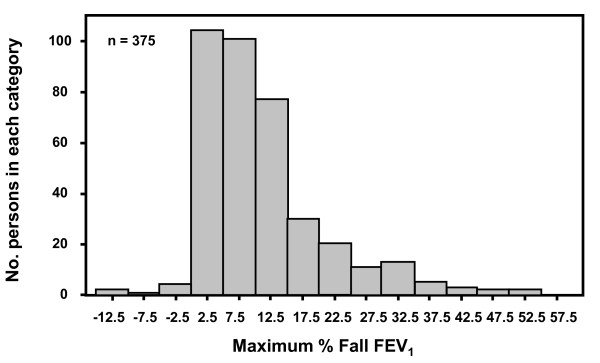
**Distribution of the maximum % fall in FEV_1_**. Distribution of the highest % fall in FEV**_1 _**after exercise challenge in 375 subjects.

Post-exercise, 163 of the 375 subjects had ≥10% fall in FEV_1 _(mean % fall ± SD was 19.1% ± 9.25 or 610 ± 330 ml) after at least one exercise challenge with 86 having ≥ 15% and 56 ≥ 20% fall in FEV_1_. Those 77 with very mild EIB i.e. 10 to 15% fall in FEV_1 _had a mean fall of 12.3% ± 1.5 or 395 ± 116 ml. The distribution of the values for the maximum % fall in FEV_1 _is given in Figure 5. Of the 163 subjects, 161 completed two exercise challenges with 88 having a fall in FEV_1 _of ≥10% at two or more time points after exercise and 157 having a fall in FEV_1 _≥ 200 ml (median 530 ml). On the first exercise challenge 119 had ≥10% fall in FEV_1_; 67 had ≥15% fall in FEV_1_. Of those 27 with a ≥12% and 200 ml after bronchodilator, 10 were positive to and 7 were negative to both exercise challenges, and 10 were positive to only one challenge.

**Figure 6:**
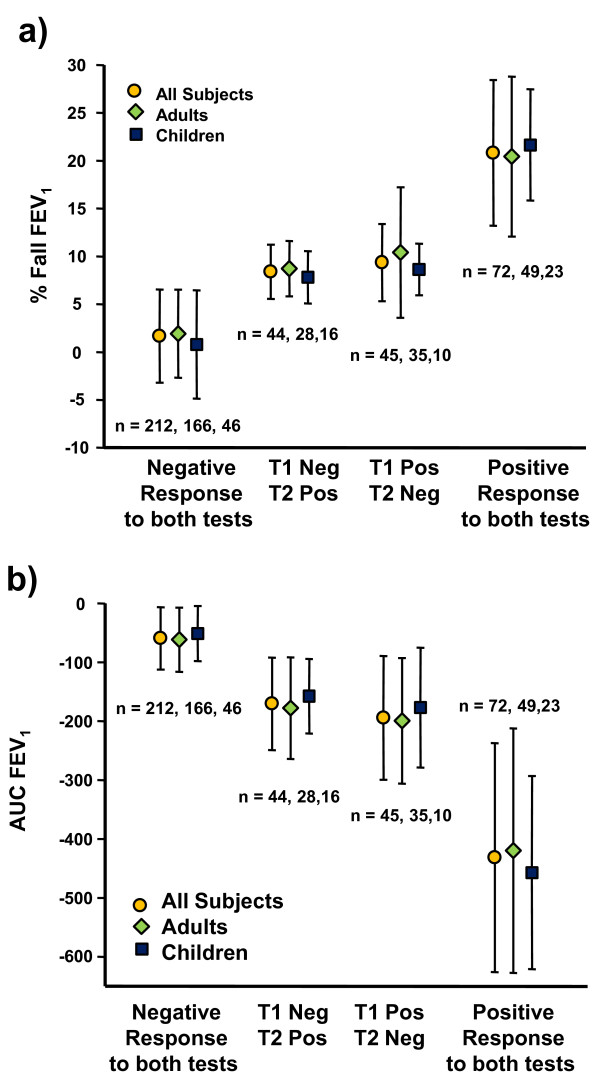
**% fall in FEV_1 _and AUC on the two exercise tests**. The mean and standard deviation for:- a) average % fall FEV_1 _on exercise; b) average AUC_0-30 min _FEV_1 _in 373 subjects and for 278 adults and 95 children. The groups are:- those negative, <10% fall in FEV_1 _after exercise, those negative/positive and positive/negative on the 1^st ^and 2^nd ^challenge, and those with two positive challenges, i.e. ≥10% fall in FEV_1_.

There were 89 subjects who had a positive test on only one of two challenges; 45 on the first challenge and 44 on the 2^nd ^challenge (Figure 6a). For the 89 the mean difference in FEV_1 _between the positive and negative test result was 308 ± 173 ml. For the 44 of 161 subjects identified as positive with a fall in FEV_1 _≥10%, only on the second challenge, 39 (89%) had a fall in FEV_1 _≤16% and only three subjects had a fall in FEV_1 _> 20%. Fifty-five of the 373 subjects had only a rise in FEV_1 _from baseline on the 1^st ^challenge; only 7 of these 55 subjects had ≥10% fall in FEV_1 _on the 2^nd ^challenge.

The mean values for % fall in FEV_1 _for adults and children and for those with two negative (< 10% fall), two positive (≥10% fall) and one positive and one negative test on each occasion are illustrated Figure 6a. AUC_0-30 min _associated with these % falls in FEV_1 _is given in Figure 6b. There was no significant difference in the response to exercise between adults and children. There was a significant correlation between the maximum % fall in FEV_1 _and the corresponding 'maximum' AUC_0-30 min _(r = 0.87, p < 0.001).

### Work Load

The exercise load was similar on both tests days. Exercise resulted in a HR, % predicted maximum at 2 and 6 minutes of 82.1% ± 5.6 and 86.6% ± 8.9 on Day 1 and of 81.5% ± 6.7 and 89.9% ± 6.5 on Day 2 in adults (p = NS) and 81.9% ± 5.7 and 85.9% ± 10.3 on Day 1 and 81.8% ± 6.3 and 86.7% ± 4.9 on Day 2 in children (p = NS). There was no significant difference in the estimated ventilation expressed as a % of maximum voluntary ventilation between Days 1 and 2 for either the adults (Day 1 at 2 min 56.8% ± 15.3 and Day 2 58.0% ± 15.2) and children (Day 1 at 2 min 54.7% ± 13.1 and Day 2 56.3% ± 11.9).

There was no significant difference in the HR, % of predicted maximum at 2 and 6 minutes on the day of the highest percent fall in FEV_1 _of 82.0% ± 5.0 and 87.4% ± 5.0 in adults, and 82.4% ± 5.1 and 86.9% ± 5.1 in children.

**Figure 7:**
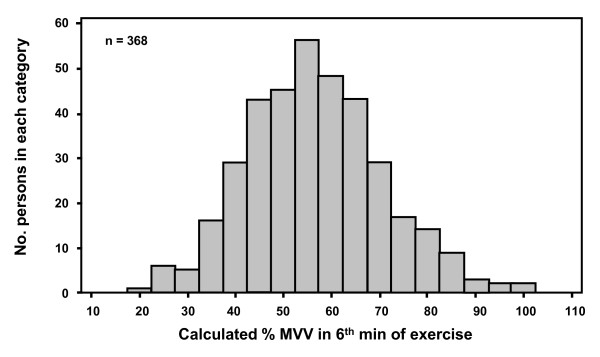
**Distribution of the % of maximum voluntary ventilation during the 6^th ^minute of exercise**. Distribution of the values estimated for percentage of maximum voluntary ventilation during exercise test on the test when the highest fall in FEV**_1 _**was measured.

The distribution of the estimated ventilation as % of MVV during the exercise is shown in Figure 7. The mean estimated ventilation calculated as a percent of maximum voluntary ventilation during the 2^nd ^and 6^th ^minute of the exercise with the highest fall in FEV_1 _was 57.3% ± 14.5 and 53.1% ± 12.9 for adults and 54.6% ± 12.9 and 51.1% ± 11.0 for the children. The estimated ventilation as % of MVV on the 2^nd ^exercise test showed a small (+1.21% MVV) though significantly (< 0.009) higher value compared with the 1^st ^test for adults and a small (+1.35% MVV) but not significantly (P < 0.052) different value for children.

There was no significant difference between the HR % predicted and estimated ventilation % MVV between the test on the day the highest % fall in FEV_1 _was documented, and on the test on the day the lowest % fall in FEV_1 _was recorded for the different groups of subjects (data not shown). There was also no significant difference in baseline FEV_1 _% predicted for the two days in the group where the % falls in FEV_1 _≥10% with both tests. The FEV_1 _% predicted was higher on the day of the highest % fall in FEV_1 _for all the other groups; however, the baseline values for FEV_1 _% predicted were always above 90% and all the differences were less than 2.4% predicted.

## Discussion

One problem in using an exercise challenge to identify EIB in the laboratory is ensuring that intensity of exercise, exercise duration, and condition of the inspired air are controlled and are adequate for eliciting the EIB response. In this multicentre study exercise duration was 8 minutes, inspired air was dry, and intensity of exercise was sufficient for HR to reach the value required by the protocol, i.e. 80-90% predicted maximum by the 2^nd ^minute of exercise and HR was not significantly different on the two test days. Appropriate times for withdrawal of medications were verified and pre-exercise FEV_1 _was >70% predicted in all but 2 subjects (both children) and it was similar on both occasions (and was actually greater than a mean of 90%). No subject had taken inhaled corticosteroids within the last 4 weeks, or long or short- acting beta_2 _agonist for 48 hours or 8 hours, respectively. Minimising the difference in these variables between tests allowed us to examine the natural variation of the airway response within a few days. We used one time point ≥10% fall to identify a positive test because this has been common practice. However we allowed a period of 5 minutes for recovery before the first FEV_1 _was measured. We excluded those who were symptomatic to the allergens to which they tested positive to a skin test at the time to reduce variability due to environmental factors. We are unaware of any other study that has given this level of attention to variables when performing two exercise challenges to identify EIB. Knowledge about normal variation in the exercise response is critically important when interpreting a negative test or when evaluating an exercise response to a therapeutic agent.

The ventilation reached and sustained during exercise is a primary determinant of the % fall in FEV_1 _[4]. However equipment for measuring ventilation during exercise is expensive and heart rate has been preferred to confirm the intensity of exercise in the United States of America. To ensure that subjects reached the minimum ventilation (40% of MVV recommended by other protocols [4]) we made an estimate of oxygen consumption from the speed and slope of the treadmill and the weight of the subject protocols and assumed a ventilatory equivalent of 27L of ventilation per L of VO_2 _using published equations [4]. This target ventilation was achieved between by the 2^nd ^minute of exercise and MVV exceeded 50% in the majority of adults and children. While a direct measurement of ventilation would have been preferable the estimated values, based on the work load and expressed as a % MVV, at 2 min and 6 min were the same as the values measured in adults during 8 minutes of bicycle exercise [25].

As may have been expected from a group of patients without a definitive diagnosis of asthma, the response to exercise, when positive, was mild and 77% of the subjects had a fall in FEV_1 _< 15% on both exercise challenges. In only 34 of 161 subjects did a ≥15% fall occur on both exercise challenges, a frequency probably consistent with their mild symptoms and indefinite diagnosis of asthma. A fall in FEV_1 _after exercise of ≥20% is the value suggested for inclusion in clinical trials to evaluate a drug for EIB (FDA Guidance for Industry, http://www.fda.gov./cder/guidance). This value occurred on two exercise challenges in only 19 of the 161 subjects (11.8%) with EIB in this study or only in 5.1% of the subjects who were exercised twice.

For those who had two exercise challenges with falls greater than 10%, the mean maximum fall after exercise was 24.7% ± 9.7, leaving little doubt about a diagnosis of EIB. The reproducibility of the response in this group was ±14.6% and compares well with the value of ±15.8% calculated in adults with an established diagnosis of asthma performing repeated exercise on a cycle ergometer [25].

We assigned a value of 0% fall for those demonstrating only a rise in FEV_1 _in response to exercise; a post-exercise fall is characteristic of asthma while a post-exercise rise in FEV_1 _is not and occurs in many non-asthmatic subjects [26]. The mean maximum fall in FEV_1 _plus 2SDs (4.9% ± SD 2.9) for the group with two negative challenges (e.g. those who had <10% fall in FEV_1 _on both challenges) was 10.7% and similar to that reported for groups of normal adults or children, without a history of symptoms of asthma, exercising in ambient air in a laboratory [2,10,27]. Thus, subjects with an NAEPPII asthma severity score of ≥1 can have a reproducible response to exercise similar to that of a healthy subject with no history of asthma

The study results confirm that there is little difference between adults and children for the indices used to express EIB and we used a value of 10% in both groups. However higher cut-off values have been recommended to identify EIB in children [28,29]. Using the 15% cut point recommended by Haby [28], the prevalence of EIB in the children was reduced from 51.5% (49/95) to 28.4% (27/95). We consider that the 5 times difference in the degree of EIB in those with ≥10% fall in FEV_1 _(24.7% ± 9.7) on both occasions and those with ≤10% fall on both occasions (4.9% ± 2.9) supports the use of a 10% cut-off to include or exclude a definitive diagnosis of EIB when challenges are repeated over a short period.

We used a cut off point of ≥10% fall in FEV_1 _to analyse the AUC_0-30 min _and its reproducibility. There was also >5 times difference in the AUC_0-30 min _between those with two challenges with ≥10% fall in FEV_1 _(-525 ± 245% FEV_1 _min^-1^) compared with those with two challenges with <10% fall in FEV_1 _(-89 ± 75% FEV_1 _min^-1^). Based on the mean plus 2SDs in those with two challenges with <10% fall in FEV_1_, we suggest an upper cut-off value for AUC_0-30 min _of 240% fall in FEV_1 _min^-1 ^for a negative test. The utility of having values for the reproducibility of AUC_0-30 min _is that there are drugs such as montelukast that have limited effect on the maximum % fall in FEV_1 _but have a profound benefit in enhancing recovery of FEV_1 _to baseline [5]. In keeping with others [30] who reported a smaller group of known asthmatic subjects over a longer period, the values for reproducibility of the % fall in FEV_1 _were superior to the AUC_0-30 min_.

In the 89 subjects positive on only one challenge (Figure 6) we considered that this variation may have been due to a change in the intensity of exercise on the two test days or perhaps other characteristics of this group. However the variation in the % fall in FEV_1 _on the two test days was not explained by differences in the ventilation % MVV, HR % predicted maximum. The FEV_1 _% predicted was significantly higher (p < 0.02) on the day of the positive challenge (92.1% ± 11.3) compared with the day of the negative test (90.2% ± 11.1) although the difference was small. The variability between a positive and negative test result may be due to other factors, perhaps environmental or dietary, or simply the intrinsic reproducibility of the test itself.

The study group had mild symptoms and signs suggestive of asthma but the NAEPPII grading could not be relied upon either to identify EIB or to predict its severity or reproducibility of the response. However, the NAEPPII is a score of asthma severity [14] and does not necessarily include symptoms provoked by exercise. This may not be important in that other investigators who have questioned subjects specifically about exercise symptoms have found symptoms alone to be unreliable predictors of either presence or severity of EIB [10,31,32].

The data presented here are a secondary analysis of a previously reported study (NCT00252291) [13]. The protocol required two exercise challenge tests to be performed under the same controlled conditions on consecutive visits prior to a mannitol and a methacholine challenge. All but two subjects of the 375 in the previously reported study performed two challenge tests. For these reasons this study offered an ideal opportunity to determine reproducibility of the response to exercise in a large group in an unbiased manner.

The usefulness of these data are not only in understanding that more than one test may be required to exclude a diagnosis of EIB but also in determining the benefit of treatment or how severe EIB should be for inclusion in a drug trial. For example the variability in the % fall in FEV_1 _as expressed by the 95% probability value for subjects with two tests ≥20% was 14.3% and the mean % fall in FEV_1 _was 30.3%. That means that on a second test a subject with a fall of 30.1% on initial testing would fall 30.1% ± 14.3% (range 44.4-15.8%) on a second occasion exercising under identical conditions within a few days. Thus for a drug to be regarded as beneficial the % fall would need to be less than 15.8% on repeated challenge.

In our subjects with mild symptoms of asthma, good lung function, and a low response rate to bronchodilator, a single exercise test did not rule out mild EIB and a second exercise test under the same conditions identified an extra 44 subjects, 27% of the total positive, with ≥10% fall in FEV_1_. It is unlikely that repeat exercise challenge is useful in those recording a rise in FEV_1 _on the initial challenge, as the chance of being positive on the second test was low and, even when the exercise challenge was positive, the falls in FEV_1 _were very mild.

## Conclusions

The majority of subjects with signs and symptoms suggestive of asthma without a definitive diagnosis will have the same outcome i.e. positive or negative test result following rechallenge when exercise is standardized for intensity, duration, and condition of the inspired air. However a minority will have a positive test result on only one exercise test. These data also show that for most subjects the EIB will be mild (< 15% fall in FEV_1_) and particularly so for those positive on a second challenge after the first exercise challenge was negative. This study provides evidence for the degree of variability in response to duplicate exercise challenges and suggests that for some subjects with mild symptoms more than one test may be required before either a diagnosis of EIB is excluded or prophylactic treatment is prescribed. Finally, these data in a large number of adults indicate that the reproducibility of the response in adults is similar to that observed in children.

## Abbreviations

AUC_0-30 min_: area under the % fall in FEV_1 _time curve; BHR: bronchial hyperresponsiveness; CDL: ClinDataLink; EIB: exercise-induced bronchoconstriction; FEV_1_: forced expiratory volume in one second; FVC: forced vital capacity; ITT: intention to treat; MVV: % of maximum voluntary ventilation; NAEPPII: National Asthma Education and Prevention Program II: NIH National Institutes of Health; PPP: per protocol population.

## Competing interests

SDA is the inventor of the mannitol test however the intellectual property is owned by her employer, the Sydney South West Area Health Service (SSWAHS). SDA receives a 10% share of the royalties paid to SSWAHS. SDA has undertaken research studies that were funded by Pharmaxis. She is a shareholder in Pharmaxis but holds no options. She acts as a consultant to Pharmaxis for which she has received fees since April 2009.

DSP, KWR, HB, CAS participated in the study through their respective centers (see below) that received a research grant for study participation from Pharmaxis Ltd.

CPP owns shares in Pharmaxis Ltd which she herself has purchased. She has also acted as a paid consultant to Pharmaxis

SN is the statistician employed by CompleWare and carried out the statistical analysis.

JW is the President of, and is a shareholder in, CompleWare Corporation. CompleWare received a fee from Pharmaxis Ltd. for services in carrying out the clinical trial.

There are no other competing interests or conflicts of interest.

## Authors' contributions

SDA & JMW designed the protocol, DSP, KWR, HB, & CAS were investigators and exercised the subjects, CP & SN carried out the statistical analysis, SDA drafted the manuscript but all of the authors contributed to the manuscript. All authors read and approved the final manuscript. 

## Authors' Information

SDA, DSP, KWR, HB & JMW have all published in the field of exercise-induced bronchoconstriction, both in adults and children, over a long period of time. They appreciated the opportunity afforded by design of the protocol standardized for the intensity and duration of exercise, and the condition of inspired air. This allowed, for the first time, a detailed analysis of reproducibility in subjects most likely to be referred to a laboratory for exercise testing to identify EIB, i.e. subjects with mild symptoms of asthma but without a definite diagnosis.

Homer Boushey is Chief of the Division of Allergy/Immunology and Director of the Asthma Clinical Research Center at the University of California.
